# The In Vitro Ability of *Klebsiella pneumoniae* to Form Biofilm and the Potential of Various Compounds to Eradicate It from Urinary Catheters

**DOI:** 10.3390/pathogens11010042

**Published:** 2021-12-31

**Authors:** Monika Oleksy-Wawrzyniak, Adam Junka, Malwina Brożyna, Migdał Paweł, Bartłomiej Kwiek, Maciej Nowak, Beata Mączyńska, Marzenna Bartoszewicz

**Affiliations:** 1 Department of Pharmaceutical Microbiology and Parasitology, Faculty of Pharmacy Wroclaw Medical University, 50-556 Wroclaw, Poland; malwinabrozyna@gmail.com (M.B.); beata.maczynska@umed.wroc.pl (B.M.); marzenna.bartoszewicz@umed.wroc.pl (M.B.); 2 Department of Environment Hygiene and Animal Welfare, Wroclaw University of Environmental and Life Sciences, 51-630 Wroclaw, Poland; pawel.migdal@upwr.edu.pl; 3 Faculty of Medicine, Lazarski University, 02-662 Warszawa, Poland; bartlomiej.kwiek@lazarski.pl; 4 Department of Drug Form Technology, Faculty of Pharmacy Wroclaw Medical University, 50-556 Wroclaw, Poland; maciej.nowak@umw.edu.pl

**Keywords:** biofilm, *Klebsiella pneumoniae*, CAUTI, antiseptics, surfactants, catheters

## Abstract

Urinary infections related to the presence of bacterial biofilm on catheters are responsible for loss of patients’ health and, due to their high frequency of occurrence, generate a significant economic burden for hospitals. *Klebsiella pneumoniae* is a pathogen frequently isolated from this type of infection. In this study, using a cohesive set of techniques performed under stationary and flow conditions, we assessed the ability of 120 *K. pneumoniae* strains to form biofilm on various surfaces, including catheters, and evaluated the usefulness of clinically applied and experimental compounds to remove biofilm. The results of our study indicate the high impact of intraspecies variability with respect to *K. pneumoniae* biofilm formation and its susceptibility to antimicrobials and revealed the crucial role of mechanical flushing out of the biofilm from the catheter’s surface with use of locally active antimicrobials. Therefore, our work, although of in vitro character, may be considered an important step in the direction of efficient reduction of *K. pneumoniae* biofilm-related hospital infections associated with the presence of urine catheters.

## 1. Introduction

**Figure 1:**
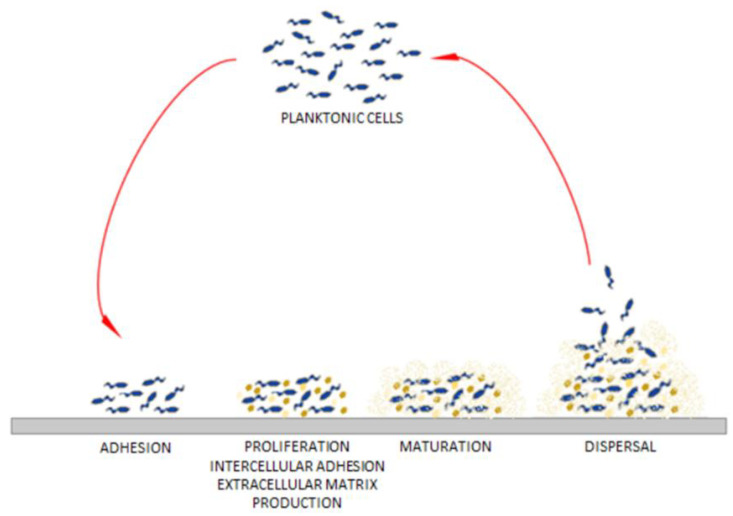
The process of biofilm formation on the adaptation from [3].

The vast majority (99%) of microbes on Earth exist in communities referred to as biofilms. These complex populations of microorganisms (of the same or different species) are embedded within a heterogeneous extracellular matrix consisting of proteins, lipids, polysaccharides and extracellular DNA (eDNA). Biofilm can float at the air-liquid interface or it can be attached to biotic or abiotic surfaces [1]. Biofilms are characterized by high tolerance to all types of disadvantageous environmental conditions. Biofilm-forming cells communicate with each other through chemical signals (e.g., by quorum sensing, QS), which allows them to simultaneously control gene expression and enables their adjustment to the environment [2]. The high cell density in a heterogeneous biofilm intensifies the exchange of genetic information, including the transmission of resistance genes between strains and species. In turn, the extracellular matrix also protects the biofilm-forming microbes from invasive species able to occupy the same ecological niche. The process of biofilm formation involves four stages: adhesion (specific and non-specific), accumulation, maturation, and dispersal (Figure 1) [3,4,5,6,7]. Due to the differential availability of oxygen in individual layers of the biofilm (and specific species requirements), some cells (known as “dormant phenotype” cells) display a reduced metabolism. The cells with slow metabolism are characterized by increased tolerance to those antimicrobials which act by inhibiting their activity [8,9]. The biofilm structure also contains dead cells in an advanced state of decomposition [1]. The eDNA, released from cells as a result of lysis, binds the extracellular matrix of the living cells, intertwines with it and increases the values of the matrix mechanical parameters.

After biofilm maturation, the dispersed aggregates or single bacterial cells are released from the biofilm matrix and migrate to search for new niches in which to settle [7].

With respect to human pathogenesis, it is commonly accepted that 80% of infections, including nosocomial ones, are related to the presence of biofilm [1], referred to in this case as “medical biofilm”. This form of microbial existence related to the macroorganism (patient) displays a high tolerance to antimicrobial agents (e.g., antibiotics, antiseptics) and to the immune system’s non-cellular (e.g., antibodies, peptides) and cellular elements (e.g., neutrophils, macrophages, leukocytes).

It is recognized that the high level of medical biofilm resistance to antimicrobials is caused by the presence of a small sub-population (approx. 1%) of surviving cells. These cells are incapable of growing in a nutrient-restricted environment and exhibit high resistance to antibiotics. They are considered to be responsible for the efficient recovery of biofilm structure, damaged by prior antimicrobial activity. This hypothesis is supported by the observation that newly formed biofilms are already highly resistant to antibiotics at a very early stage of their development [6,10].

It has been shown that ongoing exposure of biofilm-forming cells to sublethal doses of antibiotic leads to the selection of resistant cells which begin to divide and spread within the biofilm and to transmit resistance genes to the neighboring, previously sensitive, cells [11,12]. It has also been shown that such concentrations of antibiotics (often referred to as “sub-MIC”, i.e., sub-minimal inhibitory) can also stimulate the production of polysaccharide, the main component of mucus, which immobilizes and inactivate these antimicrobial agents [13]. The high accumulation of microorganisms in a relatively small space also increases the concentration of antibiotic-inactivating enzymes produced by the bacteria. Together, the above-mentioned factors result in demonstrated biofilm antibiotic resistance (or tolerance, if factors unrelated to intrinsic or acquired antibiotic resistance are considered) which is 1000 times higher compared to that of planktonic cells of the same species and strain [11,14].

Numerous devices are now commonly used in the diagnosis and treatment of nosocomial patients. They are, to a major extent, built from materials which are designed for contact with the live tissue of the patient, and hence are referred to as “biomaterials”. However, the implantation of the biomaterial by surgical intervention may contribute to the initiation of the process of microbial adhesion to the surface. Depending on the time of formation, species characteristics and environmental conditions, the size of such biofilm may range from a few microns to several millimeters in thickness. Fragments of mature biofilm may detach from the surface and migrate through blood vessels to distant parts of the body, causing generalized infections and leading to the formation of new biofilm in other locations [15,16]. The most common causes of nosocomial infections are biofilms formed on urological and vascular catheters, various kinds of endoprostheses, artificial heart valves, peritoneal dialysis catheters, as well as on artificial ventilation equipment and infusion pumps.

Urinary tract infections (UTIs) are one of the most common infections in humans. According to WHO (World Health Organization), UTIs account for 40% of all nosocomial infections and 10–20% of community-acquired infections. As much as 80% of hospital urinary infections are associated with the presence of a urinary catheter (catheter-associated urinary tract infections, CAUTI). According to statistical data, 30% of cases of bacterial UTIs lead to generalization of the inflammatory process and/or to urosepsis [17].

Before the crucial role of biofilm in the pathogenesis of UTI became known, the acquisition and spread of resistance mechanisms among urinary pathogens were considered the biggest challenge in UTI treatment. One of the most dangerous of these mechanisms is the ability of Gram-negative rods to produce enzymes inactivating β-lactams, e.g., extended spectrum β-lactamases (ESβL), metallo-β-lactamases (MβL) or *Klebsiella pneumoniae* carbapenemase (KPC). The bacteria of the genus *Klebsiella* sp. are characterized by exceptional speed in acquiring these plasmid-encoded enzymes. Moreover, *K. pneumoniae* is responsible for 6–17% of opportunistic urinary tract infections, with the majority of these related to the presence of biofilm formed inside the urinary catheter or at the catheter insertion site [18].

*K. pneumoniae* is one of the pathogens that displays a very high ability to form biofilm, which frequently takes the form of mucoid, cohesive slime. The virulence factors important for *K. pneumoniae* biofilm formation include the presence of type 1 and 3 fimbriae, Kp type fimbriae and KPF-28 fimbriae, as well as numerous non-fimbrial adhesive factors, such as the polysaccharide sheath, outer membrane lipopolysaccharide, CF29K protein and the P-like adhesive factor (with properties analogous to the P fimbriae of *E. coli*). The ability of *K. pneumoniae* to alkalize urine (by means of urease) is also of high importance in the pathogenesis of urinary tract infections [4,19].

Under physiological conditions, the urinary tract (except the urethra) remains sterile. Many factors, including the anatomical structure of the excretory system, the physicochemical properties of urine and the activity of the immune system, are responsible for maintaining the urinary tract in such a state. The most important mechanisms protecting against UTIs include the length of the urethra and the distance from it to the anus (in men), regular exfoliation of the epithelium, ureteral peristalsis, proper function of the vesicoureteral valves, acidification and concentration of urine, the presence of organic acids and nitrogen compounds, proper composition of the vaginal microbiome and antibacterial properties of prostate secretion. Mechanisms preventing the adherence of bacteria in the urinary tract, such as mechanical washing away of microorganisms during micturition, mucopolysaccharides of the bladder wall, IgG and IgA antibodies, and the presence of endogenous substances in the urine with antibacterial properties (e.g., Tamma–Horsfall protein, lactoferrin, lipocaine and antimicrobial peptides) are also considered to play a pivotal role in the process [20,21,22]. Despite the wide spectrum of these protective measures, *K. pneumoniae* UTI infections, associated with the presence of biofilm formed in the catheter, still pose a significant threat to the health of nosocomial and ambulatory patients [10,15].

The treatment of infections related to the presence of biofilm on biomaterials is a huge therapeutic challenge and an economic burden for healthcare systems and the patients themselves. Insufficient penetration of the antibiotic to the site of the infection and resistance of bacterial cells in the deeper layers of the biofilm constitute a barrier to conventional antibiotic therapy. Often, the only solution is to remove the catheter and, if possible, to replace it with a new one [23]. However, when the clinical condition of the patient does not allow removal of the implant, it is necessary to control the development of the infection. Therefore, a spectrum of locally administered and acting antimicrobial solutions was tested for this purpose. The main goal of the present research was to investigate their usefulness towards clinical, biofilm-forming *K. pneumoniae* strains in an in vitro setting, using a cohesive set of analytical techniques.

## 2. Results

### 2.1. The Ability of K. pneumoniae to Form Biofilm on Polystyrene Surface in a Standard Microbiological Medium

The aim of this experimental line was to measure the total *K. pneumoniae* biofilm biomass formed on polystyrene under stationary conditions in the standard culture medium, Tryptic Soy Broth (TSB). The crystal violet solution applied for this purpose dyes both live and dead cells as well as all components of the extracellular matrix. The performed analysis revealed that all scrutinized *K. pneumoniae* strains can form biofilm under stationary conditions on polystyrene in the applied culture medium. Of note is that different levels of biomass were recorded, underlining *K. pneumoniae* intra-species variability with respect to this parameter. The lowest absorbance value (0.398) was obtained for the Kp107 strain, while the highest (3.9168) was obtained for the Kp83 strain. The average value of absorbance obtained for 118 clinical strains of *K. pneumoniae* was 1.8479 ± 0.8267, with a median of 1.6596. Based on the data obtained, the strains (within the analyzed population) were divided into four groups:

- Ab < 1—strains with low (among the tested strains) biofilm-forming ability;
- 1 ≤ Ab < 2—strains with moderate (among the tested strains) biofilm-forming ability;
- 2 ≤ Ab < 3—strains with high (among the tested strains) biofilm-forming ability;
- Ab ≥ 3—strains characterized by very high (among the tested strains) biofilm-forming ability.

**Figure 2:**
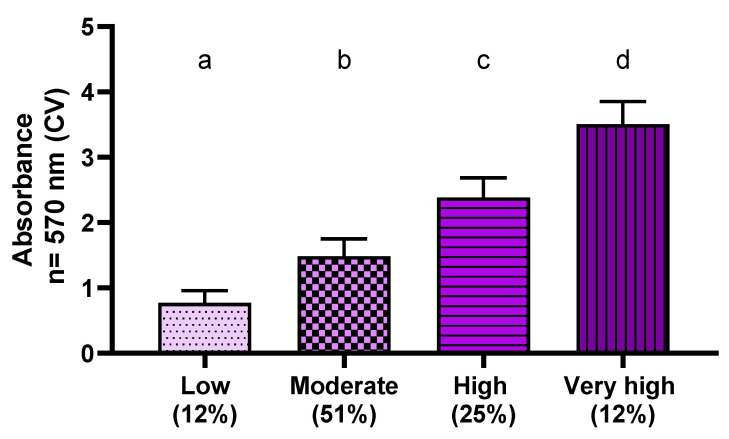
The level of average absorbance at the wavelength λ = 570 nm for each group of clinical strains of *K. pneumoniae* (Kp) biofilm biomass created on polystyrene in TSB stained with a crystal violet (CV) solution; n of strains = 118 (100%). The letters a,b,c,d express the statistically significant differences (*p* > 0.5) between four analyzed groups of *K. pneumoniae* strains. Numbers in brackets indicate percentage of strains classified into each group.

Figure 2 shows the results of average absorbance for each group of clinical *K. pneumoniae* strains at a wavelength of λ = 570 nm.

The reference strains from the American Type Culture Collection (ATCC) were applied as a control. For *K. pneumoniae* ATCC 4352 and ATCC 70063 the absorbance value was 1.32 ± 0.25 and 1. ± 0.35, respectively.

### 2.2. Semi-Quantitative Measurement of the Number of Bacterial Cells Attached to Polystyrene Surface

The technique of staining with tetrazolium salts (Richard’s method) allows indirect determination of the number of live cells adhered to the surface, by colorimetric measurement of their metabolic activity.

Based on the results of assessments of the total biofilm shown in Figure 2, 40 clinical strains, of different biofilm formation ability (10 strains of each group indicated in Figure 2), were selected.

**Figure 3:**
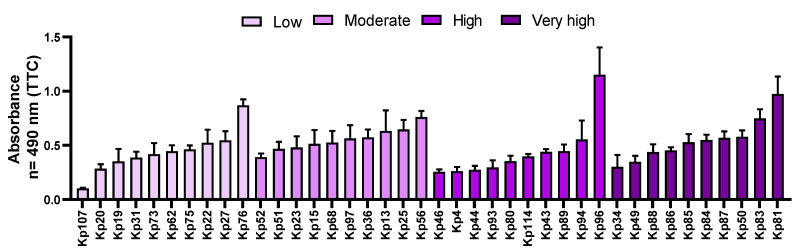
The mean values of metabolic activity of 40 *K. pneumoniae* (Kp) clinical strains, forming biofilm on polystyrene in TSB. The colors of the bars show the groups of strains with different ability to form biofilm biomass (based on the crystal violet (CV) assay results). The darker the color, the higher the ability to form biofilm biomass was recorded (please refer to Figure 2). “low”—low capability to form biofilm; “moderate”—moderate ability to form biofilm; “high and very high”—high and very high ability to form biofilm.

Among the clinical *K. pneumoniae* strains, the lowest absorbance result was obtained for the Kp107 strain (0.1026), while the highest was for the Kp96 strain (1.1522). Of note is that the Kp107 strain exhibited the lowest absorbance values not only according to the Richards’ method but also in CV assay (Figure 2 and Figure 3, respectively). The average absorbance value for all tested strains was 0.4968 ± 0.2, and the median was 0.4653. Among the strain groups with high and very high biofilm biomass forming ability, the highest value (0.9739) from the TTC method was obtained for the Kp81 strain. Differentiation in outcomes of metabolic activity was observed within the groups of strains with different ability to form biofilm biomass. As an example, the Kp81 strain formed a very strong biofilm biomass (according to the results of crystal violet dyeing) and displayed high metabolic activity (according to TTC-based results); however, while Kp76 strains’ metabolic activity was recognized as high (among the tested strains), its ability to form biofilm biomass was assessed as low. Detailed results for the clinical strains are presented in Figure 3.

**Figure 4:**
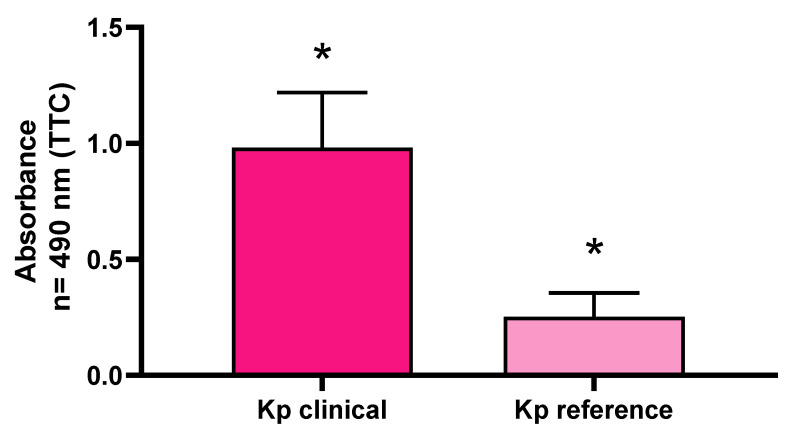
The ability of *K. pneumoniae* (Kp) clinical and reference strains to form biofilm on polystyrene surface assessed with tetrazolium chloride (TTC) staining. Statistically significant differences (*p* > 0.5) are marked with asterisks (*).

The comparison of the average absorbance between the clinical strains of *K. pneumoniae* and the reference strains showed that the strength of biofilm formation measured using the semi-quantitative method was statistically significantly higher for the clinical strains than for the reference ones (Figure 4).

### 2.3. Testing the Strength of Biofilm Formation on the Surface of a Biomaterial by Quantitative Culture Method

**Figure 5:**
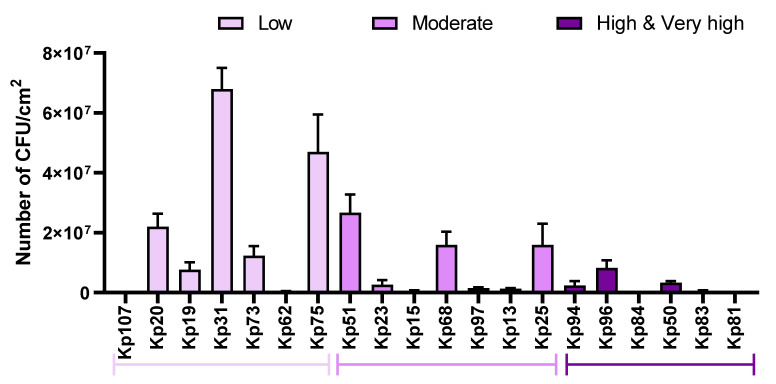
Assessment of the number of biofilm-forming cells of *K. pneumoniae* (Kp) clinical strains on the urological catheter. Strains belonging to groups with different biofilm formation ability (established based on CV results in TSB) are marked with different shades of purple. “low”—low capability to form biofilm; “moderate”—moderate ability to form biofilm; “high and very high”—high and very high ability to form biofilm.

The aim of the study was to assess the number of biofilm-forming cells on the surface of a urological catheter. Based on the results of biofilm staining with crystal violet and TTC, 20 clinical strains with differing capacity for biofilm formation (low, moderate, high, very high) were selected. The results from quantitative culturing (QC) are presented in Figure 5.

**Figure 6:**
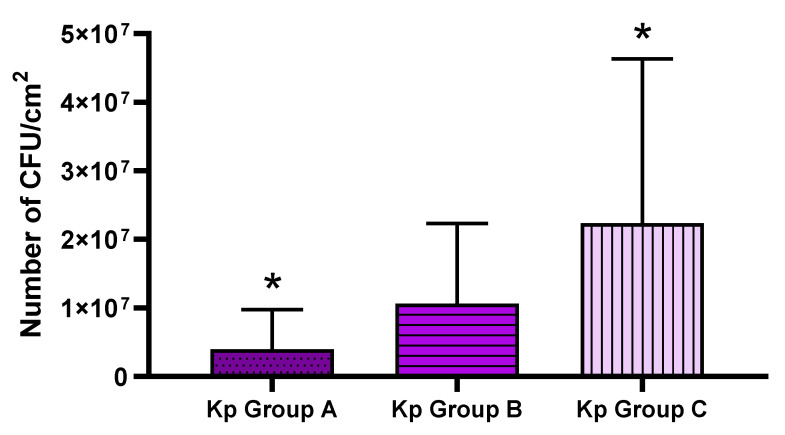
Mean values of colony forming unit (CFU)/cm^2^ obtained for three groups of clinical *K. pneumoniae* (Kp) strains of different abilities to form biofilm on polystyrene, assessed by means of quantitative culturing. Group A—strains with high and very high biofilm formation ability; Group B—strains with moderate biofilm formation ability; Group C—strains of low biofilm formation ability. Statistically significant (*p* > 0.5) differences are marked with asterisks.

The results showed that the strains recognized in the CV and Richard’s assays (Figure 2, Figure 3 and Figure 4) as having a low ability to form biofilm on polystyrene (marked in Figure 6 as “group C”) showed a higher ability (expressed as the number of biofilm-forming cells) to colonize the catheter surface made of polyvinyl chloride and, analogously, those strains which displayed a high ability to form biofilm on polystyrene in screening tests (marked in Figure 6 as “group A”) displayed a low ability to form biofilm on the polyvinyl chloride surface of catheters. The highest value of CFU/cm^2^ of catheter was obtained for the Kp31 strain (68 × 10^6^), while the lowest value was obtained for the Kp107 strain (77 × 10^4^ CFU/cm^2^ of catheter). The data on the results of the quantitative culturing of biofilm-forming *K. pneumoniae* strains are presented in Figure 6.

The difference between group A (strains of high/very high ability to form biofilm in screening tests) vs. group C (strains of low ability to form biofilm in screening tests) was statistically significant (*p* > 0.5).

### 2.4. The Ability to Form Biofilm on Polystyrene Surface in Artificial Urine Media

Based on the results of the screening tests using CV and TTC staining, two clinical strains were selected for further tests, which were performed in an artificial urine medium and in artificial urine medium supplemented with glucose. These strains were Kp81, of high biofilm formation ability, and Kp107, of low ability to form biofilm. In addition, two reference strains were applied to enable repetition of our experiments by other research teams.

**Figure 7:**
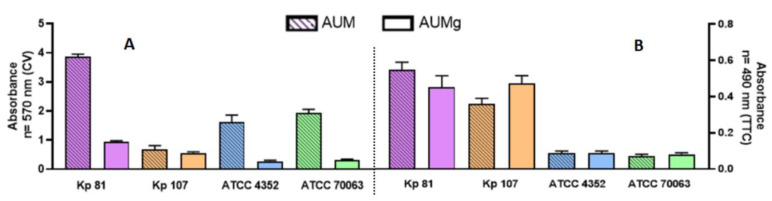
Comparison of biofilm formation ability on polystyrene by *K. pneumoniae* (Kp) reference and clinical strains in artificial urine (AUM) and artificial urine with glucose (AUMg). Analyses were performed using crystal violet CV-based assay, panel (**A**), or TTC-based assay, panel (**B**).

Significant differences in outcomes of total biomass were observed using the CV assay. In the applied artificial urine media, the clinical strain Kp107 and both reference strains produced less biofilm compared to the clinical strain Kp81. The level of biofilm biomass formed by the Kp81 strain was comparable (absorbance value of ~4), regardless of the type of medium applied (AUM, AUMg, but also TSB, as seen in previous experiments). Both reference strains formed statistically significantly less biofilm in artificial urine medium with glucose than the clinical strain Kp81 (*p* > 0.5) (Figure 7).

For the TTC-based methods, the recorded values of absorbance for both reference strains were significantly lower than those measured for both clinical strains, regardless of the type of medium applied (Figure 7).

### 2.5. Testing the Strength of Biofilm Formation on Catheter Surface in Conditions Imitating Urinary Tract Environment (Flow Conditions)

**Figure 8:**
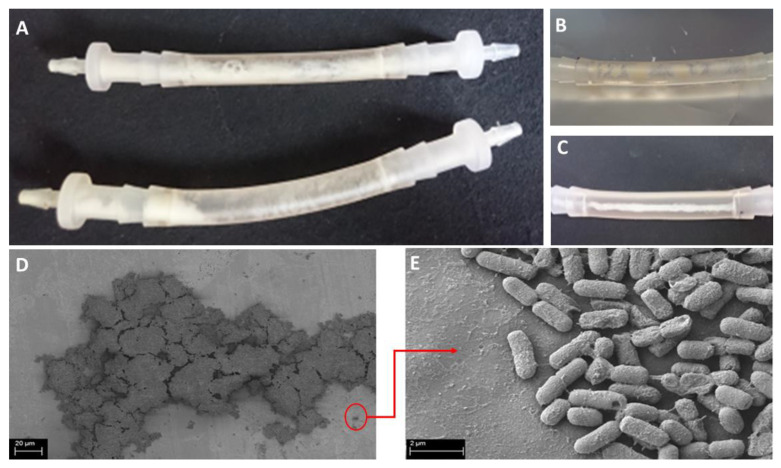
Macro-(**A**–**C**) and micro-(**D**,**E**) photographs of Kp 81 biofilm clogged inside the urinary catheter surface under flow conditions. SEM magnification: (**D**) 2900×, (**E**) 55,000×.

The experiment was carried out using the Richards’ method, both in a complete microbiological medium and in a medium imitating the urine of a diabetic patient. The flow conditions of bacterial suspensions in Tryptic Soy Broth and in artificial urine with glucose were achieved as a result of the peristaltic pump maintaining the flow rate at the level of the average glomerular filtration rate. Moreover, the presence of biofilm in the applied conditions was visualized using scanning electron microscopy (Figure 8). Of note was that the attached biofilm was present at the catheter surface in an amount visible with the naked eye (Figure 8).

**Figure 9:**
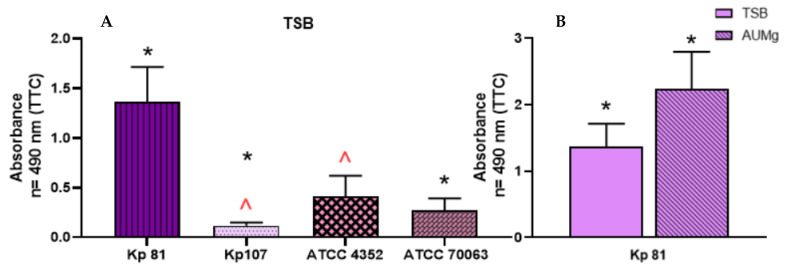
The ability of *K. pneumoniae* (Kp) clinical and reference strains to form biofilm on the urinary catheter under flow conditions in the Tryptic Soy Broth (TSB) medium (**A**) and in artificial urine medium with glucose (AUMg) (**B**) assessed with tetrazolium chloride (TTC) staining. Statistically significant differences are marked with asterisks and peaks.

The highest level of biofilm formed was observed for the clinical strain Kp81, which was also classified as a strain having a very high capacity to form a biofilm in the prior analyses performed under static conditions. The other clinical strain, Kp107, formed the weakest biofilm under flow conditions among the tested strains (Figure 9). Moreover, in the case of the Kp81 strain, a statistically significantly (*p* > 0.5) higher ability to form biofilm under flow conditions in artificial urea than in TSB was observed.

### 2.6. Evaluation of the Effectiveness of Selected Antimicrobial Substances against Biofilm Formed on the Surface of Polystyrene in Artificial Urine Enriched with Glucose

The selected antimicrobial substances (i.e., ciprofloxacin, chlorhexidine, antimicrobial peptides) and surfactants (i.e., betaine, saponin) on the *K. pneumoniae* biofilm formed in an artificial urine medium with glucose were categorized according to the level of effect exerted. Two groups of substances were distinguished: those whose activity correlated with non-complete eradication of biofilm and those whose activity correlated with complete eradication of biofilm (Table 1). Among the antimicrobial compounds tested against a clinical biofilm of *K. pneumoniae* strains, polyhexanide (PHMB) displayed the highest effectiveness. The PHMB MBEC for strain Kp107 and Kp81 was 2 mg/L and 4 mg/L, respectively. The effective concentration of chlorhexidine was determined at 4 mg/L and 8 mg/L against the biofilms of the Kp81 and Kp107 strains, respectively. The octenidine dihydrochloride completely eradicated the biofilm of the Kp81 strain at 32 mg/L, and at 64 mg/L in the case of the Kp107 strain. The application of other antimicrobial compounds (i.e., ciprofloxacin, antimicrobial peptides) did not lead to a complete eradication of biofilm (within the range of the tested concentrations). The highest reduction value observed for ciprofloxacin was 75% (in the case of the Kp81 strain) with no measurable effect towards the Kp107 strain. The antimicrobial peptide referred to as CAMEL was able to eradicate (at a concentration of 256 mg/L) 39% and 31% of the Kp81 and Kp 107 strains, respectively, while the corresponding values for citropin were 44% and 33%. The common denominator of the tested surfactants was their higher efficacy against the biofilm of the Kp81 strain than against other strains tested. The percentage value of cells remaining after treatment with saponin for the Kp81 strain was 50% and in the case of betaine it was 54%. In the case of the Kp107 strain, the application of betaine resulted in 34% cell removal. Of note is that saponin was ineffective against this strain, even at a concentration of 256 mg/L. Under the conditions of the experiment, all tested compounds were more effective against the reference *K. pneumoniae* ATCC 4352 strain than against the *K. pneumoniae* ATCC 70063 strain. Within the range of the tested concentrations, the minimum concentrations eradicating biofilm were determined for all antiseptics used in the experiment as well as for the antibiotic ciprofloxacin.

The lowest MBEC value was found for polyhexanide at 2 mg/L against both reference strains. The same MBEC value was found for ciprofloxacin against the Kp4352 strain, while a higher concentration of this antibiotic was effective against the Kp70063 strain (8 mg/L). Chlorhexidine was effective against the biofilm of the Kp4352 strain at a concentration of 4 mg/L, while against the biofilm of the Kp70063 strain, the required concentration was 8 mg/L. Among the antiseptics, octenidine showed the highest (i.e., the least favorable) MBEC values. A concentration of 32 mg/L and 64 mg/L was needed for complete eradication of the Kp4352 and Kp70063 biofilms, respectively.

The use of the CAMEL peptide at the highest tested concentration resulted in a reduction of the biofilm of the Kp4352 strain of 88% (the survival rate was therefore 12%), while in the case of the Kp70063 strain, the biofilm survival rate was 44%. The use of the second tested peptide, citropin 1.1, at a concentration of 256 mg/L, resulted in 47% eradication, i.e., survival of 53% of the biofilm formed by the Kp4352 strain and survival of 73% of the biofilm formed by *K. pneumoniae* ATCC 70063. Surfactants (i.e., betaine and saponin), on the other hand, even at the maximum tested concentration, had no effect on the biofilm formed by the standard Kp70063 strain, while partially reducing the biofilm of the strain Kp4352. Survivability in the presence of 256 mg/L of saponin was at the level of 51% compared to the control. The same concentration of betaine reduced the biofilm formed by the Kp4352 strain by only 20%.

### 2.7. Evaluation of the Effectiveness of Selected Antiseptic and Surfactant Compounds against the Biofilm Formed under Flow Conditions on the Surface of a Urinary Catheter

**Figure 10:**
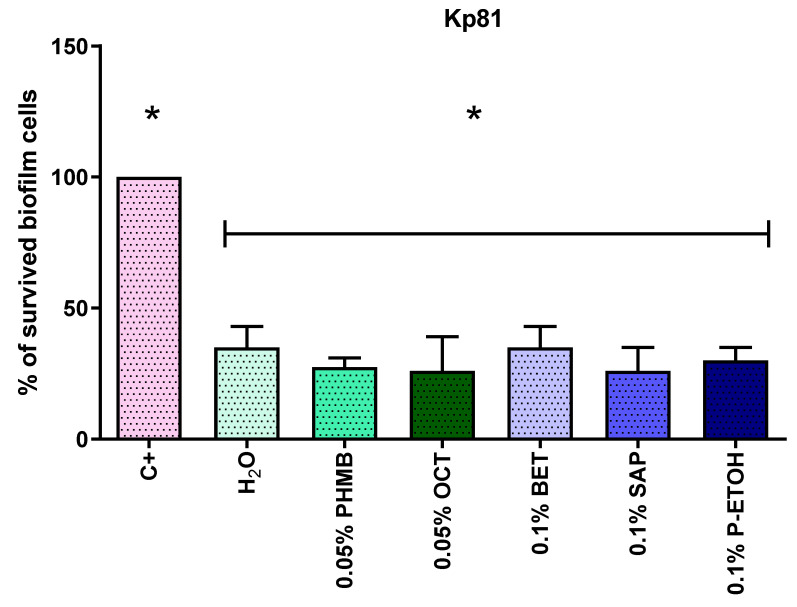
Comparison of the effectiveness of selected compounds in the eradication of *K. pneumoniae* clinical strain K81 biofilm from the catheter surface under flow conditions. C+, control of growth; H_2_O, sterile water; PHMB, polyhexanide; OCT, octenidine; BET, betaine; SAP, saponin; P-ETOH, phenoxyethanol. Statistically significant differences are marked with asterisks.

The aim of this experimental line was to evaluate the efficacy of selected compounds (with efficacy against biofilm formed in stationary conditions proven in earlier stages) against biofilm formed under flow conditions. The clinical strain Kp81, with the highest biofilm-forming ability, was included in this study. The comparison of the obtained results is presented in Figure 10.

Comparison of the survival results of the biofilm formed on the catheters under flow conditions after treatment with antiseptic compounds (polyhexanide, PHMB; octenidine, OCT), surfactants (betaine, BET; saponin, SAP; phenoxyethanol, P-ETOH) and water, showed a statistically significantly lower survival than the control for all tested substances. However, no statistically significant differences between the individual substances were found. Regardless of whether the catheter was rinsed with water, antiseptic at a concentration similar to a practical one, or a detergent, biofilm survival compared to control was below 50%.

## 3. Discussion

The scope of this study included two sequential experimental stages. First, the biofilm formation ability of clinical and reference *K. pneumoniae* strains was tested depending on the culture conditions and the adhesion surface. As a first step, screening was performed on a large (*n* = 120) pool of strains to select representative strains for further research. The second part of the research focused on the potential for eradication of *K. pneumoniae* biofilm (formed under different conditions) by means of various methods. The studies under stationary conditions were performed with reference strains and two clinical strains with the highest and lowest biofilm formation capacity (determined by prior CV, TTC and QC analyses). The assays under flow conditions were carried out for the selected clinical strain that showed the highest biofilm formation ability among all the strains tested.

The first screening study aimed to measure the total biomass of the biofilm formed by the test species under optimal growth conditions. The dye used, crystal violet, stains all the components of the biofilm, both live and dead cells, as well as the extracellular matrix. The absorbances at the wavelength λ = 570 nm for the 118 tested clinical strains and two reference strains ranged from 0.398 to 3.9168. It was shown that all the microorganisms tested had the ability to form biofilm. Based on the results of the absorbance of crystal violet (CV) staining of the biofilm formed in the complete culture medium under stationary conditions, it was decided to divide the tested strains into four groups with different biofilm formation abilities (Figure 2). The crystal violet staining technique is the primary screening method for sessile biofilm detection. Due to the simplicity of its implementation, it is widely used by many researchers [24,25,26,27]. Bellifa et al. showed a significant percentage of strains strongly forming biofilm (69%) when assessing the ability of *K. pneumoniae* to form biofilm on polystyrene. However, the absorbance values obtained by the research team were much lower than the results obtained in the present study (the highest value obtained in the Bellifa et al. study was 2.5 [28]). Of note is that due to various methodological disadvantages (especially when slime-forming biofilms are analyzed), the crystal violet staining method is considered an initial, often required, but preliminary technique to assess the level of biofilm biomass. However, Allkja et al. showed that the method, using crystal violet and microtiter plates, may be useful even in interlaboratory comparative trials. There it requires adapting of the protocols at the reading stage, especially in terms of drawing individual calibration curves in each of the laboratories [29].

Biofilm is a compact, spatial community of interacting bacterial cells surrounded by an extracellular matrix, with a specific level of metabolic activity. Staining with a 2,3,5-triphenyltetrazolium chloride solution measures the metabolic activity of live biofilm-forming cells, so the absorbance result depends not only on the number of living cells in the sample, but also on their metabolic activity. A representative pool of 40 strains was selected for the study, which represented four groups with different biofilm formation abilities, based on their CV staining results. The average absorbance levels at the wavelength λ = 490 nm were statistically significantly higher for the clinical strains than for the reference strains. It should be noted that the metabolic results (TTC assay) were differentiated within groups of different biofilm biomass formation ability (CV assay). In each of the groups, distinguished based on crystal violet (CV) staining, there were strains both with a low ability to form biofilm measured by TTC staining, as well as those with a high ability for biofilm formation (Figure 3). The distribution of results was similar in each group. Therefore, it can be concluded that the high absorbance results in staining with the TTC solution of strains considered to be weak biofilm-formers (measured by CV assay) may have resulted from the high metabolic activity of the adhered cells or from a phenomenon in which biofilm was formed by a high number of cells with a low content of extracellular matrix. By analogy, strains identified as strong biofilm-formers by means of CV, which display low absorbance levels in a TTC assay, may have a low level of cellular metabolism or form biofilm consisting of a relatively low number of cells producing a relatively high amount of the extracellular matrix. Other research teams have assessed cell viability based on the reduction of tetrazolium salt compounds equally often as based on staining of the biofilm with a crystal violet solution [30]. Due to the possibility of detecting only metabolically active cells, the above-mentioned method is readily chosen for assessing the effectiveness of antimicrobial agents [31]. Despite the limitations resulting from the required specific culture conditions (e.g., appropriately fertile medium, oxygen supply), the Richards’ method using TTC staining is considered more sensitive and specific in detecting biofilm than staining with crystal violet [32].

Urinary tract infections are one of the most common hospital infections. Patients with diabetic nephropathy are particularly at risk. Due to its composition, urine promotes the multiplication of bacteria. However, it should be noted that it differs from the rich, standard microbiological media. Two clinical strains were selected to assess the ability of *K. pneumoniae* to form biofilm in a medium simulating physiological human urine and the urine of diabetic patients—a strain with high biofilm-forming ability and a strain with low biofilm-forming ability (selected based on CV and TTC results). The obtained results indicated that the strain with a previously detected high biofilm-forming ability in a standard medium, produced statistically significantly more biofilm biomass on the polystyrene surface compared to the strain with a weak ability to form biofilm (Figure 7). The assessment of biofilm by indirect measurement of the number of adhered cells showed that in the media imitating physiological urine and urine of diabetic patients, clinical *K. pneumoniae* strains form biofilm more strongly compared to the reference *K. pneumoniae* strains (Figure 7). However, our experiment did not demonstrate that the addition of glucose has a significant effect on the increase in the amount of metabolically active cells. In their studies of 2013, Paganelli et al. found an increased biofilm production by *Enterococcus faecium* strains grown in a medium enriched with glucose. However, their measurement results were based on crystal violet staining [33,34]. Other researchers have found growth stimulation of the pathogens responsible for most UTIs when cultured in media simulating human urine [35,36].

The results of the screening tests prompted an attempt to assess the strength of biofilm formation by the tested species on the surface of a urinary catheter made of polyvinyl chloride. The applied quantitative cultures method showed an inverse correlation of the results obtained in the biomass staining screening tests of the formed *K. pneumoniae* biofilm on polystyrene with the actual number of bacterial cells adhering to the catheter surface (Figure 6). Strains with high and very high biofilm-forming ability on polystyrene showed a relatively low average number of cells attached to polyvinyl chloride, in contrast to strains that showed weak ability to form biofilm in screening tests. This suggests a significant role of the extracellular matrix in biofilm formation by bacteria of the *K. pneumoniae* species, especially that the results of quantitative culturing of two representative strains (Kp81, of high biofilm-forming ability in screening tests, and Kp107, with weak biofilm-forming ability) indicated the relatively low number (in comparison to other strains) of cells colonizing the catheter surface (Figure 5). Results obtained by other researchers confirm the usefulness of quantitative cultures as a simple, accurate and repeatable method of assessing biofilms formed on the surface of catheters [37,38].

To reflect the interactions between the microorganisms and the patient’s urinary catheter, the appropriate experimental setting was established. Our research, carried out under flow conditions, tested the ability of the investigated microorganisms to form biofilm in the environment of a flowing liquid imitating the urine of a catheterized patient. Despite the constant movement of the artificial urine at a speed resembling the physiological one, the microorganisms were able to adhere to the internal walls of the catheter. Within the next 24 h they produced an attached biofilm in an amount detectable with the naked eye (Figure 8). The spatially diverse structure of the biofilm formed inside the lumen of the catheters was also detected by means of SEM (Figure 8). The assessment of the amount of biofilm formed under flow conditions indicated that the strain showing high biofilm-forming ability (assessed by screening methods) under flow conditions also formed a stronger biofilm than a representative strain displaying weak ability to form biofilm. This indicates the dependence of the level of adhesion on the type of surface. High absorbance values, obtained despite the constant movement of cells and washing them through the medium, may indicate a high presence of adhesion factors.

The study of biofilm formed under conditions simulating the urinary tract environment (i.e., flow in artificial urine medium with glucose) showed stronger biofilm formation abilities of the tested strains than those determined under standard laboratory conditions. In the case of strong biofilm producers (e.g., strain Kp81), the results were up to twice as high as in the test in an artificial urine medium with glucose and under flow conditions for PVC catheter than under stationary conditions on polystyrene in TSB (Abs_490_: 2.47 vs. 0.97) and up to five times higher than in the study of the stationary plate microtiter culture of AUMg (Abs_490_: 2.47 vs. 0.45). These results suggest that the features of the applied biofilm model should be scrutinized before any conclusions concerning the strain’s actual ability to form biofilm in clinical conditions can be drawn. Additionally, the observed strong biofilm formation on the surface of the polyvinyl chloride biomaterial suggests that PVC is not the optimal material for production of urinary catheters. This suggestion is supported by results presented by other authors. In 2010, when comparing the strength of biofilm formation on the surface of catheters made of various materials, Mączyńska et al. showed that *K. pneumoniae* strains isolated from various types of infections adhered strongly to the surface of the Nelaton’s catheter [39].

Biofilm is a community of microorganisms with extremely high resistance/tolerance to antimicrobial agents. The antibiotics routinely used in therapy are considered too large molecules to effectively penetrate the lower layers of the biofilm and they are often retained by the structure of the extracellular matrix. Acting only on the external layers of the biofilm, such antibiotics may cause a risk of emergence of microbial resistance. The messenger molecules being components of the bacterial communication system (e.g., QS, quorum sensing), transmit information about the presence of an antibiotic (stressor factor) to the cells located in the deeper layers of the biofilm, so that the remaining cells activate their stress-answer systems. Therefore, for local treatment and prevention from material-related infection, especially in the case of urinary catheters, the application of antiseptics is recommended. These are products registered for the local treatment of chronic wounds, as well as for the care of surgical wounds, injection sites or urinary catheters. Antiseptics are considered much more effective than antibiotics against biofilm-forming microorganisms. Among known substances with antimicrobial activity, surface-active substances, such as detergents or plant and animal biosurfactants, usually used in combination with antiseptics, deserve attention [7]. Eight compounds from different groups were selected to assess their ability to eradicate biofilm under stationary conditions. They were ciprofloxacin (chemotherapeutic agent), betaine and saponin (detergents), CAMEL and citropin 1.1 (antimicrobial peptides) and polyhexanide, octenidine and chlorhexidine (antiseptics).

Chemotherapeutic agents were represented by ciprofloxacin which has a bactericidal mechanism with a wide substrate spectrum and can be very readily used in various types of infections, with a particular emphasis on urinary tract infections (UTIs).

The choice was determined, among others, by the wide availability of the drug, good pharmacokinetic and pharmacodynamic parameters and, last, but not least, EUCAST recommendations for UTI therapy with Gram-negative bacilli etiology [40]. MBEC values were only established for the reference *K. pneumoniae* strains and were relatively high. According to the breakpoints in the EUCAST tables (MIC breakpoints ≤ 0.25 mg/L), these strains should be considered resistant ones [40]. However, it should be remembered that the values in the EUCAST tables are determined by methods other than those applied in this study and should not be compared directly or, if necessary, should be compared with caution. For the clinical multi-resistant *K. pneumoniae* strains, the highest therapeutically effective concentration of ciprofloxacin tested resulted in only a 25% biofilm reduction recorded for the strain of high biofilm-forming ability. The obtained results showed that, despite therapeutic recommendations, ciprofloxacin is not the best drug in the treatment of UTI infections, considering that these are infections related to the presence of biofilm. Reports on the development of resistance of the *Enterobacteriaceae* to this antibiotic focus mainly on its effectiveness against planktonic forms of microorganisms. As previously mentioned, the biofilm structure is a special form of microbial growth which is characterized by increased resistance/tolerance to antimicrobial substances. It should also be considered that drug susceptibility tests routinely performed in laboratories are carried out in the environment of complete culture media, and the microbial cells growing in the form of the so-called turf have a morphotype intermediate between planktonic and biofilm forms [41].

In the experiments described in this paper, with respect to detergents, saponin and betaine solutions were used. In high concentrations, these compounds act by violating the integrity of the outer membranes of the microbial cells and, in lower concentration (as they are applied in antiseptics), they act mainly as surface active agents (surfactants). Tambone et al. also described the ability of natural surfactants to dissolve extracellular mucus [42]. None of the surfactants tested in the present study, in the analyzed concentration range, was able to completely remove the biofilm. It is important, however, that in practice, surfactants are used for treatment in combination with topically acting antimicrobial agents. Studies carried out by Romanelli et al. confirm the positive effect of the combination of betaine and polyhexanide on the course of wound healing [43]. Both substances show synergism—betaine disturbs the matrix structure, while PHMB acts directly on pathogen cells. Similar observations were also made by Davis et al. [44].

None of the applied antimicrobial peptides were sufficiently effective in the tested concentration range against the biofilm of the investigated strains. However, in recent studies (2018), Jaśkiewicz et al. showed the high effectiveness of citropin 1.1 and CAMEL against planktonic forms of microorganisms, which translated into the possibility of using these compounds in preventive management. Research on the design of new antimicrobial substances with a protein structure should therefore constitute an important element in the strategy of searching for effective solutions in the fight against biofilm [45].

Antiseptics showed the highest activity against the biofilm of the tested *K. pneumoniae* strains. MBEC values were determined for all three substances (PHMB, OCT, CHX), but for polyhexanide and chlorhexidine they were significantly lower than for octenidine. Polyhexanide caused a complete reduction of the biofilm of all tested strains, at a concentration lower than that of other antiseptics. Importantly, all the studied antiseptics caused a complete reduction of the biofilm of all test strains at concentrations from several to several hundred times lower than the concentrations used in medicinal preparations available on the market. Studies from 2019 by Henly et al. on the effectiveness of various biocides against the uropathogenic biofilm of *Escherichia coli* showed the highest effectiveness for polyhexanide among other tested compounds [46]. Thus, the obtained results confirm the effectiveness of antiseptics against the biofilm of the examined strains, formed under stationary conditions on polystyrene. Moreover, the antibiotic effective concentrations of these compounds are significantly lower than the therapeutic concentrations.

Due to the growing resistance to antibiotics, properly applied topical antimicrobials are still considered effective and safe. In view of the results obtained, they seem to be the best weapon in the fight against biofilm-forming pathogens. However, when choosing a substance for therapy, one should consider not only the effectiveness and antimicrobial spectrum of the substance, but also possible adverse reactions that may occur after contact with tissues. It is necessary to know the current safety data sheet of the medicinal product, which lists the indications of the registered use, as well as the emerging tolerance of microorganisms to low concentrations of these compounds.

Particular attention should be paid to the danger of using chlorhexidine and octenidine on biofilm formed by *Pseudomonas aeruginosa* rods. Although these bacteria are not the most common cause of UTIs, they show an unusual ability to form biofilm and are widespread in the hospital environment and may acquire tolerance or even resistance to the above-mentioned compounds. Efflux pumps of *P. aeruginosa* actively remove the chlorhexidine (CHX) inside the bacterial cell and are responsible for the failure of attempts to eradicate the biofilm. The above CHX pump-out mechanism may also occur in *Acinetobacter baumannii*. By actively removing CHX from inside the cell, efflux pumps also cause cross-resistance to ciprofloxacin and aminoglycosides [47]. Additionally, in the case of chlorhexidine, one should be aware of possible anaphylactic reactions, including shock [48].

Octenidine dihydrochloride is an antiseptic with a high biocompatibility index and has, until recently, been rated as one of the most effective against biofilm forms of microorganisms. In 2018, Shepherd et al. observed the development of tolerance of *P. aeruginosa* and its ability to adapt in an environment of increasing antiseptic concentrations. Although the tested concentrations were lower than the useful concentrations of antiseptics, the acquired tolerance was maintained in subsequent passages of the strain without the presence of the antiseptic. Additionally, the studies concerned planktonic cells, which are much more sensitive to the action of antimicrobial substances [49]. The observed phenomenon is a signal for continuous monitoring of the sensitivity of microorganisms to octenidine and for strict control of the use of octenidine-based preparations in departments where the risk of *P. aeruginosa* infection is particularly high.

Polyhexanide is the only one of the three tested antiseptics to which no emerging resistance, tolerance or reduction in the sensitivity of microorganisms has yet been observed. Polyhexanide was characterized by the highest effectiveness against the microorganisms tested in biofilm form and the MBEC values for polyhexanide were lower than for other antiseptics. The consensus of Kramer et al., published in 2018, confirms the effectiveness of antiseptics against biofilm forms of microorganisms and recognizes these compounds as the first and the most important element in the effective prevention of biofilm formation in wounds and in the prevention of infections associated with the development of already formed biofilm. It also indicates that a combination of surfactants with locally active antimicrobials (e.g., PHMB with betaine) is characterized by high anti-biofilm effectiveness [50].

When determining the sensitivity of biofilms to antimicrobial agents, one should decide whether the value determined in the test relates to the lowest concentration inhibiting time-dependent biofilm development (i.e., MBIC, minimal biofilm inhibitory concentration) or to the minimum concentration needed for a complete eradication of an already formed biofilm (MBEC) [51].

While searching for the possibilities of preventing and treating urinary tract infections related to the formation of biofilm on the catheter surface, it was necessary to test the effectiveness of known antimicrobial compounds in conditions that occur in vivo. The clinical strain of *K. pneumoniae* selected for the study, based on the results of screening tests, was recognized as the strain with the highest biofilm formation capacity, Kp81. The obtained results showed no statistically significant differences in the reduction of biofilm between the substances used. The use of antiseptics, surfactants and water together resulted in below 50% survival of the biofilm on the catheter surface. One should note the differences in the results obtained for polyhexanide and octenidine compared to those for the biofilm tested under stationary conditions on polystyrene surface. Although the study of the effectiveness of antiseptics against biofilm under stationary conditions was conducted at much lower concentrations (the highest tested was 0.0256%), MBEC values were also obtained. It is worth noting that the test under stationary conditions was carried out in artificial urine medium, while the test under flow conditions was carried out in a complete culture medium. The MBEC values were determined in the range of PHMB and OCT tested concentrations in a stationary study on polystyrene, in the environment of an artificial urine medium with glucose (which can stimulate the proliferation of bacterial cells). However, in the flow test with the use of urological catheter and Tryptic Soy Broth, twice the concentration of the antiseptics resulted in a reduction of only several dozen percent. Considering the above, it can be hypothesized that the effectiveness of the antiseptics also depends on the environment and that in artificial urine medium with glucose they evidence a better effect. This may suggest that routine antimicrobial susceptibility testing in microbiology laboratories produces results which are overestimates, and therefore that antimicrobials may be more effective in vivo than the results obtained in vitro show. On the other hand, despite the insignificant differences between the reduction of biofilm under the influence of water and under the influence of the tested compounds, one can assume that the demonstrated reduction could have occurred due to the mechanical flushing of the lumen of the biofilm-clogged catheter. The lack of differences between flushing with water and the tested solutions may have resulted from too short a contact of the tested antimicrobials with the cells. It should also be remembered that in the assessment of the ability to form biofilm in a full culture medium under flow conditions, the Kp81 strain used for the study on a polyvinyl chloride catheter formed biofilm nearly three times more strongly than under stationary conditions on polystyrene in a culture with artificial urine medium with glucose. Considering the above, the lower efficiency of rinsing the catheter with water and the tested substances with antimicrobial activity in biofilm eradication compared to the results of research on the effectiveness of compounds under stationary conditions may also have resulted result from differences in the strength of biofilm formation depending on the surface of contact, nutrients of the medium and culture conditions. In this study, no statistically significant differences were found between the antiseptics’ ability to eradicate biofilm from catheters under flow conditions. However, Brill et al. found a statistically significantly higher level of reduction of biofilm formed on the catheter after rinsing with polyhexanide solution compared to the control, which was a catheter flushed with NaCl. The obtained results may suggest that the combination of mechanical biofilm removal by flushing the lumen of a catheter with a locally active antimicrobial is effective [52,53].

We are aware that our study would gain additional value if it was enriched with analysis of genes responsible for biofilm-mediated drug resistance. Nevertheless, at this stage of our study, we decided to investigate (with use of a cohesive set of techniques) the high number of *K. pneumoniae* strains (120 in total) to obtain detailed insight into their ability to form biofilm and intra-species variability with respect to this. Therefore, we focused on observed phenomena and not on the molecular mechanisms standing behind them. We plan to analyze the expression of relevant genes responsible for biofilm formation and tolerance of their structures to applied biocides in the next phase of investigation. Nevertheless, our in vitro work, may be considered an important step in the direction of the efficient reduction of biofilm-related, hospital, infections associated with the presence of urine catheters.

## 4. Materials and Methods

The study included 118 clinical strains of *K. pneumoniae* (Kp1–Kp118) and 2 reference strains from American Type Culture Collection (ATCC): *K. pneumoniae* ATCC 4352 (Kp4352) and *K. pneumoniae* ATCC 70063 (Kp70063). The clinical strains were isolated from patients hospitalized in 2014–2015 at the Hematology and Oncology Departments of the 1st Public Teaching Hospital in Wroclaw, during another project, approved by the Bioethical Committee of Wroclaw Medical University, protocol # September 2016. All of them presented a high level of antibiotic resistance and produced β-lactamases with an extended substrate spectrum (ESβL) or metallo-β-lactamases (MβL). The species affiliation was confirmed using the Vitek 2 Compact (BioMerieux, Warsaw, Poland) diagnostic panels assessing the biochemical properties of the isolated bacteria. Drug susceptibility and resistance mechanisms were assessed using the Kirby–Bauer disk diffusion method. A detailed susceptibility profile and isolation materials are provided in the Supplementary Materials Section. Strains were stored deep frozen at −80 °C (low temperature freezer, Thermo Electron Corporation, Waltham, MA, USA) in Tryptic Soy Broth (TSB) (Becton Dickinson, Warsaw, Poland) with 15% glycerol (POCH/Avantor, Gliwice, Poland) until analysis. Before each experiment, the microorganisms were inoculated into TSB and on MacConkey Agar (MC) (Becton Dickinson, Warsaw, Poland) to check the purity of the stored bacterial cultures. Cultures were incubated (incubator, Advantage-Lab GmbH, Darmstadt, Germany) under aerobic conditions at 37 °C for 24 h.

### 4.1. Crystal Violet

The strains were incubated in Tryptic Soy Broth, the rich composition of which contains all the ingredients necessary for their growth and provides an optimal environment for multiplication. The method with crystal violet (CV) solution, which stains both bacterial cells and the extracellular biofilm matrix produced by them, was used to assess the total biofilm biomass formed.

A suspension of 1 MF (McFarland) in a densitometer (Densimat, BioMerieux, Warsaw, Poland) was made from a 24-h culture in Tryptic Soy Broth (Becton Dickinson, Warsaw, Poland) and then it was diluted 1000-fold. One hundred milliliters of the prepared suspension of each of the test strains was applied in 6 replications to a 96-well polystyrene titration plate (Sarstedt, Blizne Laszczynskiego, Poland) and incubated for 24 h/37 °C (Incubator, Advantage-Lab, GmbH, Darmstadt, Germany). After the incubation the suspension was gently withdrawn from each well and the wells were washed 3 times with 0.9% NaCl solution (Stanlab, Lublin, Poland) to remove unadhered cells. The plate was dried at 37 °C for 10 min. In the next step, 100 µL of crystal violet solution (Aqua-med., Lodz, Poland) was added to each well. Incubation was carried out for 10 min. at room temperature. Then, the dye was stripped off and the plate was rinsed 3 times with 0.9% NaCl again and dried at 37 °C for 10 min. In the last step, 100 µL of a freshly prepared 30% acetic acid solution (POCH/Avantor, Gliwice, Poland) was added into the wells and the plate was shaken (Microplate shaker, IKA Schuttler MTS-4, IKA, Königswinter, Germany) at 400 RPM (revolutions per minute) for 10 min. The absorbance measurement was performed in a spectrophotometer (Multiskan GO, Thermo Scientific, Waltham, MA, USA) for a wavelength of λ = 570 nm.

### 4.2. Richards’ Method

The Richards’ method was used to semi-quantify the number of cells in the biofilm biomass formed on the polystyrene surface. The method used the ability of metabolically active microorganisms to reduce colorless 2,3,5-triphenyl tetrazolium chloride (TTC) to red formazan.

Suspensions with a density of 1 MF (Densitometer, Densimat, BioMerieux, Warsaw, Poland) were prepared from fresh 24-h liquid bacterial cultures in TSB (Becton Dickinson, Warsaw, Poland) and then diluted 1000-fold. In the next step, 100 µL of the prepared suspension of each of the tested strains was added to the 6 wells of a 96-well polystyrene plate (Sarstedt, Blizne Laszczynskiego, Poland) and incubated for 24 h at 37 °C (Incubator, Advantage-Lab, GmbH, Darmstadt, Germany). After incubation, the suspension was withdrawn from each well and the plate was washed 3 times with 0.9% NaCl solution (Stanlab, Lublin, Poland) to remove residual planktonic cells. Then, 100 µL of 0.1% TTC (Merck, Darmstadt, Germany) in TSB solution was added to each well and shaken for 5 min. (400 RPM) (Microplate shaker, IKA Schuttler MTS-4, IKA, Königswinter, Germany) to increase the availability of TTC to the cells. The prepared plate was incubated for 2 h at 37 °C. After that time, the reaction mixture was withdrawn, and the wells were filled with 100 µL of methanol (VWR, Radnor, PA, USA) and shaken again at 400 RPM for 10 min. After the cell walls were destroyed by methanol and formazan was released into the solution, the entire contents of the wells were transferred to the wells of a new assay plate. The absorbance measurement was performed for the wavelength λ = 490 nm in a spectrophotometer (Multiskan GO, Thermo Scientific, Waltham, MA, USA).

### 4.3. Quantitative Culture Method

The ability of microorganisms to form biofilm structures depends on the microorganism itself, the nutrients available in the environment as well as on the type of surface to which the cells adhere. The amount of biofilm formed on fragments of the Nelaton urological catheter under stationary conditions was assessed using the quantitative culture method.

Half-centimeter pieces of sterile catheter (GalMed, Bydgoszcz, Poland) were placed in wells of 24-well polystyrene plates (Sarstedt, Blizne Laszczynskiego, Poland). Then, 2 mL of bacterial suspension with a density of 10^5^ CFU/mL (colony forming unit) in TSB (Becton Dickinson, Warsaw, Poland) was added to the wells with catheters. The prepared plates were incubated in an incubator (Advantage-Lab, GmbH, Darmstadt, Germany) for 24 h/37 °C. After incubation, the catheter pieces were gently washed 3 times with 0.9% NaCl (Stanlab, Lublin, Poland) and the excess suspension was drained on a sterile gauze pad. The tested biomaterial fragments were transferred into tubes containing 1.5 mL of 0.5% saponin (Merck, Darmstadt, Germany) and shaken intensively on a vortex shaker (Microspin FV-2400, BioSan, Jozefow, Poland) for 1 min. Then, 100 µL of the obtained cell suspension was taken and its serial dilutions were created in NaCl in the range of 10^−1^–10^−9^. One hundred milliliters of each dilution was applied onto MacConkey Agar (Becton Dickinson, Warsaw, Poland) and distributed evenly. The plates were incubated at 24 h/37 °C and then the number of grown colonies was counted. The experiment was carried out in 3 technical replications.

### 4.4. The Ability of K. pneumoniae Strains to Create Biofilm on Polystyrene in Artificial Urine Medium and in Artificial Urine Medium with Glucose

The aim of the experiments was to evaluate the ability of the tested microorganisms to create biofilm on a polystyrene surface in artificial media imitating normal human urine and the urine of diabetic patients.

Experiments conducted:

- measurement of the total biomass of biofilm formed on the surface of polystyrene in artificial urine medium (AUM) and in artificial urine medium + glucose (AUMg)—crystal violet staining method,
- semi-quantitative evaluation of the number of live biofilm cells adhered to polystyrene in artificial urine and in artificial urine medium with glucose—Richards’ method.

The analyses were performed analogously to those in point Section 4.1. and Section 4.2.

The composition of artificial urine is presented in Table 2.

AUMg additionally contains 0.155% glucose (Chempur, Piekary Slaskie, Poland). Both AUM and AUMg were adjusted to pH 6.5 with 3 M hydrochloric acid (Chempur, Piekary Slaskie, Poland) and then sterilized by vacuum filtration (Filters, Thermo Fisher Scientific, Waltham, MA, USA). The tests were performed in 6 replications.

### 4.5. The Ability to Form Biofilm on Catheter Surface under Flow Conditions

**Figure 11:**
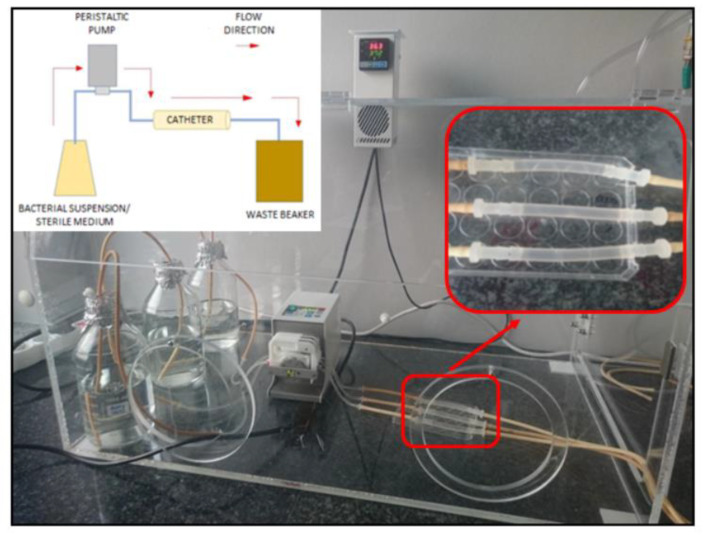
Flow test system. Sterile artificial urine medium with glucose (AUMg) flowing through catheters with *K. pneumoniae* biofilm cells.

To imitate the conditions following patient catheterization, a flow test system was constructed. It consisted of a peristaltic pump (Ismatec, Cole-Parmer GmbH, Wertheim, Germany) and a series of sterile urological catheters (GalMed, Bydgoszcz, Poland). The task of the pump was to move the fluid through the lumen of the catheter at a speed corresponding to the rate of final urine formation, taking into account the rate of glomerular filtration and subsequent resorption of primary urine in the renal tubules—0.5 mL/min. (pump setting: 1.65). The system was located in an incubator where a constant temperature of 37 °C was maintained. The structure of the system is presented in Figure 11. The aim of the experiment was to evaluate the ability of *K. pneumoniae* strains to create biofilm on the surface of the catheter under flow conditions, in a complete microbiological medium and in conditions simulating the urinary tract environment. Tryptic Soy Broth and artificial urine medium with glucose were used.

The strains were grown for 24 h/37 °C in TSB (Becton Dickinson, Warsaw, Poland) or AUMg. The density of the bacterial suspension was densitometrically adjusted to 3 × 10^8^ CFU/mL (Densimat, BioMerieux, Warsaw, Poland). The prepared suspension was passed through 7-cm fragments of the Nelaton’s catheter for 2 h at a speed similar to the glomerular filtration rate—0.5 mL/min. After 2 h, a sterile solution of 50% TSB (Becton Dickinson, Warsaw, Poland) in 0.9% NaCl (Stanlab, Lublin, Poland) or sterile AUMg, respectively, was connected to the test system.

### 4.6. Scanning Electron Microscopy

Scanning electron microscopy was used to evaluate the structure of the biofilm formed under flow conditions on the catheter surface. Fragments 2 and 6 of selected catheters from the tests carried out in point 2.5. were placed in 25% glutaraldehyde (Chempur, Piekary Slaskie, Poland) solution and stored at 4 °C until analysis time. Then, the samples were dehydrated using a standard alcohol series procedure [55]. After dehydration the samples were sputtered (Leicasputter, Leica Microsystems, Wetzlar, Germany) in Au: Pd: 60:40 mixing followed by microscopic visualization (Auriga 60, Zeiss, Oberkochen, Germany).

### 4.7. The Effectiveness of Selected Antimicrobials against Biofilm Formed on Polystyrene Microplates

Microorganisms in the form of biofilms are characterized by a particularly high level of resistance to chemical antimicrobial agents. Among the known substances with antimicrobial activity are antibiotics, antiseptics, detergents (surfactants) and peptides. The aim of the experiments was to evaluate the efficacy of exemplary compounds of these groups against the *K. pneumoniae* biofilm. The evaluation was carried out by determining the minimum biofilm eradication concentration (MBEC). The antimicrobial activity of the following compounds was determined in AUMg on polystyrene microtiter plates (Sarstedt, Blizne Laszczynskiego, Poland):

- Ciprofloxacin (CIPRO) (Merck, Darmstadt, Germany),
- CAMEL (CAM) (Lipopharm, Gdansk, Poland),
- Citropin 1.1 (CIT) (Lipopharm, Gdansk, Poland),
- Saponin (SAP) (Merck, Darmstadt, Germany),
- Betaine (BET) (Merck, Darmstadt, Germany),
- Chlorhexidine (CHX) (Fagron Pharma Cosmetics, Rotterdam, The Netherlands),
- Octenidine (OCT) (Dishman Pharmaceuticals & Chemicals Ltd., Ahmedabad, India),
- Polyhexanide (PHMB) (Carbosynth Ltd., Compton, Newbery, UK).

A bacterial suspension was prepared from a 24-h liquid culture and the density was adjusted densitometrically (Densimat, BioMerieux, Warsaw, Poland) at 1.5 × 10^8^ cells/mL (0.5 MF). Then the suspension was diluted 1000-fold in AUMg to the density of 1.5 × 10^5^ cells/mL. One hundred milliliters of the obtained suspension was transferred to the wells (1–10 and 12) of the titration plate (Sarstedt, Blizne Laszczynskiego, Poland). One hundred milliliters of sterile AUMg (K-, negative control—medium sterility) was added to the wells in column 11. The plate was incubated at 24 h/37 °C in an incubator (Advantage-Lab GmbH, Darmstadt, Germany). On the next day, the suspensions were removed, and the plate was washed 3x with sterile 0.9% NaCl (Stanlab, Lublin, Poland) to remove unadhered planktonic bacterial cells. In the next step, 100 µL of the tested substances dilutions in a geometric progression were added to the wells in columns 1–10 (Table 3). One hundred milliliters of sterile AUMg was added to the wells in columns 11 and 12.

The plate was left at 24 h/37 °C. After that time, all suspensions were removed, and the wells were gently washed 3 times with sterile saline again. The next step was the addition of 100 µL of 0.1% TTC (Merck, Darmstadt, Germany) in TSB (Becton Dickinson, Warsaw, Poland) to each well. The plate was incubated for 2 h/37 °C and then all wells were emptied. Finally, 100 µL of methanol (Chempur, Piekary Slaskie, Poland) was added into the wells and the plate was shaken for 5 min. at 400 RPM (Microplate shaker, IKA, Schuttler MTS-4, IKA, Königswinter, Germany MTS 4). The contents of all wells were quantitatively transferred to the wells of a new plate and the absorbance was measured at λ = 490 (Multiskan GO, Thermo Scientific, Waltham, MA, USA). Readout of MBEC values was performed by relating the absorbance of the tested substances dilution wells to the absorbance of the positive growth control wells (C +, column no 12).

### 4.8. The Effectiveness of Selected Antiseptic and Surfactants against the Biofilm Formed under Flow Conditions on Urinary Catheter Surface

The experiment used the flow test system described in point 4.5. A suspension with a density of 3 × 10^8^ CFU/mL was made from a 24-h culture in a Tryptic Soy Broth (Becton Dickinson, Warsaw, Poland). The suspension was flowed through the urinary catheter fragments (GalMed, Bydgoszcz, Poland) for a period of 2 h at a speed corresponding to the glomerular filtration rate—0.5 mL/min. After 2 h, a sterile solution of 50% TSB (Becton Dickinson, Warsaw, Poland) in 0.9% NaCl (Stanlab, Lublin, Poland) was flowed through the tested catheter fragment for another 24 h. After this time, solutions of the tested substances or sterile water were flowed through the catheters for 2 min. In the next step, the catheters were cut into 1 cm pieces. The 3rd, 4th and 5th fragment of the catheter were assessed using the Richards’ method. A catheter, which had not been washed with any solution after the biofilm forming step, served as a control. The efficacy of 0.05% solutions of polyhexanide (PHMB) (Carbosynth, Ltd., Compton, Newbery, UK) and octenidine (OCT) (Dishman Pharmaceuticals & Chemicals Ltd., Ahmedabad, India) and 0.1% solutions of betaine (BET) (Merck, Darmstadt, Germany), saponin (SAP) (Merck, Darmstadt, Germany) and phenoxyethanol (P-ETOH) (Merck, Darmstadt, Germany) was tested.

### 4.9. Statistical Analysis

GraphPad Prism 5 (GraphPad Software, Inc., San Diego, CA, USA) was used for statistical analysis. The Kruskal–Wallis test with Dunnett’s post-hoc analysis was used for calculations. The level of statistical significance was at *p* < 0.0001.

## Figures and Tables

**Table 1:** The effectiveness of the tested antimicrobials against *K. pneumoniae* biofilm formed on the surface of polystyrene in an artificial urine medium with glucose (AUMg). Minimal biofilm eradication concentration (MBEC) values are expressed in mg/L; the % values represent the survival rate at 256 mg/L; X indicates no effect of the maximum tested concentration.

Antimicrobial/Strain	Kp81	Kp107	ATCC 4352	ATCC 70063
Polyhexanide	44	2	2	2
Chlorhexidine	4	8	4	8
Octenidine	32	64	32	64
Ciprofloxacin	75%	X	2	8
Betaine	54%	66%	80%	X
Saponin	50%	X	51%	X
CAMEL	61%	69%	12%	44%
Citropin 1.1	56%	67%	53%	73%

**Table 2:** Composition of artificial urine medium [54].

Amount (g) *	Substance
5.2	Sodium chloride (Stanlab, Lublin, Poland)
0.37	Calcium chloride (VWR, Radnor, PA, USA)
1.3	Ammonium chloride (Chempur, Piekary Slaskie, Poland)
3.2	Sodium sulfate (VWR, Radnor, PA, USA)
0.49	Magnesium sulfate (VWR, Radnor, PA, USA)
0.0012	Iron (II) sulfate (VWR, Radnor, PA, USA)
0.07	Uric acid (Acros Organics)
0.4	Citric acid (VWR, Radnor, PA, USA)
0.1	Lactic acid (VWR, Radnor, PA, USA)
0.8	Creatinine (Acros Organics)
10	Urea (VWR, Radnor, PA, USA)
2.1	Sodium carbonate (VWR, Radnor, PA, USA)
0.95	Potassium dihydrogen phosphate (VWR, Radnor, PA, USA)
1.2	di-Potassium phosphate (VWR, Radnor, PA, USA)
1	Peptone L37 (Oxoid Ltd., Hempshire, UK)
0.005	Yeast extract (VWR, Radnor, PA, USA)

* amount per liter of water.

**Table 3:** Series of dilutions of the tested antimicrobials.

Column No	1	2	3	4	5	6	7	8	9	10
Concentration (mg/L)	0.5	1	2	4	8	16	32	64	128	256

## Data Availability

The data presented in this study are collected in repository disc and can be shared on reasonable request sent to the corresponding authors.

## Supplementary Materials

The following are available online at https://www.mdpi.com/article/10.3390/pathogens11010042/s1, Table S1: detailed susceptibility profile and isolation materials of tested clinical *K. pneumoniae* strains.
