# Supplementary material for: The In Vitro Ability of Klebsiella pneumoniae to Form Biofilm and the Potential of Various Compounds to Eradicate It from Urinary Catheters
Source: Pathogens. 2021 Dec 31;11(1):42. doi: 10.3390/pathogens11010042 (PMC8780725; doi:10.3390/pathogens11010042)
Supplement: Supplementary file 1 [file pathogens-11-00042-s001.zip › pathogens-1470516-supplementary.pdf]

Table S1. Susceptibility profiles of analyzed *K. pneumoniae* strains. S - Susceptible, standard dosing regimen’ I - Susceptible, increased exposure; R – Resistant; x - no data

Susceptibility profile and isolation materials of tested clinical *Klebsiella pneumoniae* strains

Strains No	Mechanism of Antibiotic Resistance	Sample	Amikacin	Tobramicin	Gentamicin	Amoxicillin/Clavulanic Acid	Ampicillin/Sulbactam	Ciprofloxacin	Ceftazidime	Cefotaxime	Cefuroxime	Aztreonam	Piperacillin/Tazobactam	Cefepime	Trimethoprim/Sulfamethoxazole
Kp1	ESβL	Blood	S	S	S	R	R	S	R	R	R	R	R	R	R
Kp2	ESβL	Stool	S	S	S	R	R	S	R	R	R	R	R	R	R
Kp3	ESβL	Stool	I	R	R	R	R	R	R	R	R	R	R	R	R
Kp4	ESβL	Stool	I	R	I	R	R	S	R	R	R	R	R	R	R
Kp5	ESβL	Blood	R	R	R	R	R	R	R	R	R	R	S	S	R
Kp6	ESβL	Stool	I	R	R	R	R	S	R	R	R	R	S	S	R
Kp7	ESβL	Stool	I	R	R	R	R	S	R	R	R	R	S	S	R
Kp8	ESβL	Blood	R	R	R	R	R	S	R	R	R	R	R	R	R
Kp9	ESβL	Stool	I	I	R	R	R	S	R	R	R	R	R	R	R
Kp10	ESβL	Stool	R	R	R	R	R	S	R	R	R	R	R	R	R
Kp11	ESβL	Vagina	I	R	R	R	R	R	R	R	R	R	R	R	R
Kp12	ESβL	Stool	R	R	R	R	R	S	R	R	R	R	R	R	R
Kp13	ESβL	Stool	I	R	R	R	R	R	S	R	R	R	R	R	R
Kp14	ESβL	Stool	I	R	R	R	R	R	R	R	R	R	R	R	R
Kp15	ESβL	Stool	I	R	R	R	R	R	R	R	R	R	R	R	R
Kp16	ESβL	Stool	R	R	R	R	R	R	R	R	R	R	R	R	R
Kp17	ESβL	Urine	R	R	R	R	R	R	R	R	R	R	R	R	R
Kp18	ESβL	Stool	I	R	R	R	R	R	R	R	R	R	R	R	R
Kp19	ESβL, MDR	Blood	I	R	R	R	R	R	R	R	R	R	R	R	R
Kp20	ESβL	Urine	S	S	S	R	R	S	R	R	R	R	R	R	R
Kp21	ESβL	Stool	S	S	S	R	R	S	R	S	R	R	R	S	S
Kp22	ESβL	Stool	S	S	S	R	R	R	R	R	R	R	R	R	R
Kp23	ESβL, MDR	Stool	I	R	R	R	R	R	R	R	R	R	R	R	R
Kp24	ESβL	Stool	S	S	S	R	R	S	I	I	R	R	R	I	R
Kp25	ESβL	Stool	S	S	S	R	R	S	R	R	R	R	R	R	R
Kp26	ESβL	Stool	I	S	S	R	R	R	R	R	R	R	R	R	R
Kp27	ESβL	Blood	R	R	R	S	R	S	R	R	R	R	S	R	R
Kp28	ESβL	Stool	I	R	R	R	R	R	R	R	R	R	R	R	R
Kp29	ESβL	Stool	I	R	R	R	R	R	R	R	R	R	R	R	R
Kp30	ESβL	Stool	R	R	R	R	R	R	R	R	R	R	R	R	R
Kp31	ESβL	Blood	I	R	R	R	R	R	R	R	R	R	R	R	R
Kp32	ESβL	Vagina	I	R	S	R	R	R	R	R	R	R	R	R	R
Kp33	ESβL	Urine	I	R	S	R	R	R	R	R	R	R	R	R	R
Kp34	ESβL	Stool	I	R	R	R	R	R	R	R	R	R	R	R	R
Kp35	ESβL	Urine	I	R	R	R	R	R	R	R	R	R	R	R	R
Kp36	ESβL	Urine	S	S	S	R	R	S	R	R	R	R	R	R	R
Kp37	ESβL	Urine	I	R	R	R	R	R	R	R	R	R	R	R	R
Kp38	ESβL	Stool	I	R	R	R	R	R	R	R	R	R	R	R	R
Kp39	ESβL	Stool	I	R	R	R	R	R	R	R	R	R	R	R	R
Kp40	ESβL	Urine	S	S	S	R	R	S	R	R	R	R	R	R	R

Kp41	ESβL	Urine	I	R	R	R	R	R	R	R	R	R	R	R	R	S	S	S	S
Kp42	ESβL	Stool	I	R	R	R	R	R	R	R	R	R	R	R	R	S	S	S	S
Kp43	ESβL	Urine	I	R	R	R	R	R	R	R	R	R	S	R	R	S	S	S	S
Kp44	ESβL	Stool	I	R	S	R	R	R	R	R	R	R	R	R	R	I	S	R	R
Kp45	ESβL	Stool	S	S	S	R	R	S	R	R	R	R	R	R	R	S	S	S	S
Kp46	ESβL	Vagina	I	R	R	R	R	R	R	R	R	R	R	R	R	S	I	R	R
Kp47	ESβL	Urine	I	R	R	R	R	R	S	S	R	R	R	I	R	S	S	S	R
Kp48	ESβL	Vagina	I	R	R	R	R	R	R	R	R	R	R	R	R	S	S	S	S
Kp49	ESβL	Vagina	I	R	R	R	R	R	R	R	R	R	R	R	R	S	R	R	R
Kp50	ESβL	Stool	I	R	R	R	R	S	R	R	R	R	R	R	R	S	S	S	S
Kp51	ESβL	Stool	S	S	S	R	R	I	R	R	R	R	R	R	R	S	S	S	S
Kp52	ESβL	Foreskin	I	R	R	R	R	R	R	R	R	R	R	R	R	S	S	S	S
Kp53	ESβL, MDR	Stool	I	R	R	R	R	R	R	R	R	R	R	R	R	S	R	R	R
Kp54	ESβL	Stool	I	R	S	R	R	R	R	R	R	R	R	R	R	S	S	S	S
Kp55	ESβL, Mβ	Stool	I	R	S	R	R	R	R	R	R	R	R	R	R	I	S	R	R
Kp56	ESβL	Vagina	S	R	R	R	R	R	R	R	R	R	R	R	R	S	S	S	S
Kp57	ESβL	Foreskin	I	R	R	R	R	S	R	R	R	R	R	R	R	S	S	S	S
Kp58	ESβL	Stool	I	R	R	R	R	R	R	R	R	R	R	R	R	S	S	S	S
Kp59	ESβL	Blood	I	R	S	R	R	R	R	R	R	R	R	R	R	S	S	S	S
Kp60	ESβL	Sputum	S	R	R	R	R	R	R	R	R	R	R	R	R	S	I	R	R
Kp61	ESβL	Stool	R	R	R	R	R	R	R	R	R	R	R	R	R	R	S	S	R
Kp62	ESβL	Sputum	R	R	R	R	R	R	R	R	R	R	R	R	R	S	I	R	R
Kp63	ESβL	Sputum	S	R	R	R	R	R	R	R	R	R	R	R	R	S	I	R	R
Kp64	ESβL	Stool	R	R	S	R	R	R	S	R	R	R	R	R	R	S	S	R	R
Kp65	ESβL	Throat	R	R	R	R	R	R	R	R	R	R	R	R	R	S	R	x	R
Kp66	ESβL	Throat	S	R	R	R	R	R	R	R	R	R	R	R	R	S	R	R	R
Kp67	ESβL	Stool	S	R	S	R	R	R	R	R	R	R	R	R	R	S	S	S	S
Kp68	ESβL	Stool	R	R	R	R	R	R	R	R	R	R	R	R	R	S	S	S	S
Kp69	ESβL	Stool	R	R	S	x	x	R	x	x	x	x	R	R	R	S	I	R	R
Kp70	ESβL	Stool	R	R	R	R	R	R	R	R	R	R	R	R	R	R	R	R	R
Kp71	ESβL	Stool	S	R	R	R	R	R	R	R	R	R	R	R	R	S	S	S	S
Kp72	ESβL	Urine	I	R	R	x	x	R	R	x	x	x	R	R	R	S	S	S	S
Kp73	ESβL	Stool	R	R	R	R	R	R	R	R	R	R	R	R	R	S	I	R	R
Kp74	ESβL	Stool	R	R	R	R	R	R	R	R	R	R	R	R	R	S	S	S	S
Kp75	ESβL	Stool	I	R	R	R	R	R	R	R	R	R	R	R	R	S	S	S	S
Kp76	MDR	Stool	I	R	R	R	R	R	R	R	R	R	R	R	R	R	R	R	R
Kp77	MDR	Stool	I	R	R	R	R	R	R	R	R	R	R	R	R	S	I	R	R
Kp78	ESβL	Stool	I	R	R	R	R	R	R	R	R	R	R	R	R	S	S	x	I
Kp79	ESβL	Nose	I	R	R	R	R	R	R	R	R	R	R	R	R	S	S	S	S
Kp80	ESβL	Stool	I	R	R	R	R	R	R	R	R	R	R	R	R	S	S	S	S

Kp81	ESβL	Stool	I	R	R	R	R	R	R	R	R	R	R	R	R	S	S	S	S
Kp82	ESβL	Stool	I	R	R	R	R	R	R	R	R	R	R	R	R	S	S	S	S
Kp83	ESβL	Stool	I	R	R	R	R	R	R	R	R	R	R	R	R	S	S	S	S
Kp84	ESβL	Stool	I	R	R	R	R	R	R	R	R	R	R	R	R	S	S	S	S
Kp85	ESβL	Stool	I	R	R	R	R	R	R	R	R	R	R	R	R	S	S	S	S
Kp86	ESβL	Stool	I	R	R	R	R	R	R	R	R	R	R	R	R	S	I	R	R
Kp87	ESβL	Urine	I	R	R	R	R	R	R	R	R	R	R	R	R	S	S	S	S
Kp88	ESβL, MDR	Stool	I	R	R	R	R	R	R	R	R	R	R	R	R	I	I	R	R
Kp89	ESβL	Stool	I	R	R	R	R	R	R	R	R	R	R	R	R	S	S	S	S
Kp90	ESβL	Stool	I	R	I	R	R	R	R	R	R	R	R	R	R	S	S	S	S
Kp91	MDR	Blood	R	R	R	x	x	R	R	R	R	R	R	R	R	S	I	R	R
Kp92	ESβL	Urine	I	R	R	R	R	R	R	R	R	R	R	R	R	S	S	S	S
Kp93	ESβL	Throat	I	R	R	R	R	R	R	R	R	R	R	R	R	S	S	S	S
Kp94	ESβL	Stool	I	R	R	R	R	R	R	R	R	R	R	R	R	I	R	R	R
Kp95	ESβL	Stool	I	R	R	R	R	R	R	R	R	R	R	R	R	S	S	S	S
Kp96	ESβL	Stool	I	R	R	R	R	R	R	R	R	R	R	R	R	S	S	S	S
Kp97	ESβL	Anus	I	R	R	R	x	R	R	R	R	x	R	R	R	I	I	R	R
Kp98	ESβL	Stool	I	R	R	R	x	R	R	R	R	x	R	R	R	S	S	x	R
Kp99	ESβL	Urine	I	R	R	R	R	R	R	R	R	R	R	R	R	S	S	S	S
Kp100	ESβL	Stool	I	R	R	R	R	R	R	R	R	R	R	R	R	S	S	S	S
Kp101	ESβL	Stool	I	R	R	R	R	R	R	R	R	R	R	R	R	S	S	S	S
Kp102	ESβL	Stool	I	R	R	R	R	R	R	R	R	R	R	R	R	S	S	S	S
Kp103	ESβL	Blood	I	R	S	R	R	R	R	R	R	R	R	R	R	S	S	S	S
Kp104	ESβL	Stool	R	R	R	R	R	R	R	R	R	R	R	R	R	I	I	R	R
Kp105	ESβL	Stool	I	R	R	R	R	R	S	R	R	x	R	S	R	S	S	x	R
Kp106	ESβL	Stool	R	R	R	R	R	R	R	R	R	x	R	R	R	S	I	x	R
Kp107	MDR	Sputum	S	S	R	R	R	R	R	R	R	R	R	R	R	R	R	R	R
Kp108	ESβL	Throat	R	R	R	R	R	R	R	R	R	R	R	R	R	S	S	S	S
Kp109	MDR	Stool	I	R	R	R	R	R	S	I	R	R	R	R	R	S	S	x	R
Kp110	ESβL	Stool	I	R	R	R	R	R	R	R	R	R	R	R	R	S	S	S	S
Kp111	ESβL	Urine	I	R	R	R	R	R	R	R	R	R	R	R	R	S	S	S	S
Kp112	ESβL	Urine	I	R	R	R	R	R	R	R	R	R	R	R	R	S	S	S	S
Kp113	ESβL, KPC	Stool	I	R	R	R	R	R	R	R	R	R	R	R	R	S	I	R	R
Kp114	ESβL	Urine	I	R	R	R	R	R	R	R	R	R	R	R	R	S	S	S	S
Kp115	ESβL	Stool	I	R	R	R	R	R	R	R	R	R	R	R	R	S	S	S	S
Kp116	ESβL	Stool	I	R	R	R	R	R	R	R	R	R	R	R	R	S	S	S	S
Kp117	ESβL	Stool	I	R	R	R	R	R	R	R	R	R	R	R	R	S	S	S	S
Kp118	ESβL	Stool	I	R	R	R	R	R	R	R	R	R	R	R	R	S	S	S	S
